# Transformation and Evaluation of Cry1Ac+Cry2A and *GTGene* in *Gossypium hirsutum* L.

**DOI:** 10.3389/fpls.2015.00943

**Published:** 2015-11-12

**Authors:** Agung N. Puspito, Abdul Q. Rao, Muhammad N. Hafeez, Muhammad S. Iqbal, Kamran S. Bajwa, Qurban Ali, Bushra Rashid, Muhammad A. Abbas, Ayesha Latif, Ahmad A. Shahid, Idrees A. Nasir, Tayyab Husnain

**Affiliations:** Centre of Excellence in Molecular Biology, University of the PunjabLahore, Pakistan

**Keywords:** *Agrobacterium*, Cry1Ac, Cry2A, Cp4 epsps, cotton, GTGene, *Bacillus thuringiensis*

## Abstract

More than 50 countries around the globe cultivate cotton on a large scale. It is a major cash crop of Pakistan and is considered “white gold” because it is highly important to the economy of Pakistan. In addition to its importance, cotton cultivation faces several problems, such as insect pests, weeds, and viruses. In the past, insects have been controlled by insecticides, but this method caused a severe loss to the economy. However, conventional breeding methods have provided considerable breakthroughs in the improvement of cotton, but it also has several limitations. In comparison with conventional methods, biotechnology has the potential to create genetically modified plants that are environmentally safe and economically viable. In this study, a local cotton variety VH 289 was transformed with two Bt genes (Cry1Ac and Cry2A) and a herbicide resistant gene (cp4 EPSPS) using the *Agrobacterium* mediated transformation method. The constitutive CaMV 35S promoter was attached to the genes taken from *Bacillus thuringiensis* (Bt) and to an herbicide resistant gene during cloning, and this promoter was used for the expression of the genes in cotton plants. This construct was used to develop the Glyphosate Tolerance Gene (GTGene) for herbicide tolerance and insecticidal gene (Cry1Ac and Cry2A) for insect tolerance in the cotton variety VH 289. The transgenic cotton variety performed 85% better compared with the non-transgenic variety. The study results suggest that farmers should use the transgenic cotton variety for general cultivation to improve the production of cotton.

## Introduction

Cotton is grown around the world, mostly in tropical and subtropical areas. Cotton is used in numerous ways, such as for food and animal feed and in the textile industry. Its seeds are also used in many countries for the production of cooking oil and as a livestock feed because they are a rich source of protein (Hari, 2007; Keshamma et al., 2008; John, 2011). Lint is the source of high quality fibers. Cotton fiber plays a very important role in the manufacture of different products, such as textile products and paper (Akhtar et al., 2004). The cultivation of cotton is reported in more than 50 countries around the world, including China, America, India, Brazil, and Pakistan. The approximate area for the cultivation of cotton is 10,000 hectares per year worldwide (Hari, 2007; Azam et al., 2013). However, 87% of this cotton cultivation area is located in developing countries (Keshamma et al., 2008), which means that only 13% of the total area is located in developed countries. Cotton is considered “white gold” because of its importance to the economy of Pakistan. (Pakistan Bureau of Statistics, 2013–14).

In addition to the importance of cotton, cotton cultivation faces many problems, such as insect pests, weeds and viruses. In the past, insects were controlled by insecticides, but this caused economic loss in the country. Although conventional breeding methodology has made significant progress in the field of cotton improvement, it has limitations to its ability to introduce new alleles (Keshamma et al., 2008). This method still cannot solve the problem of insecticides. To combat losses from insects and pests, insecticides are used excessively every year in developing countries, such as Pakistan. Biotechnology has the potential to create new plants, new genes and new products that are environmentally safe and economically viable (John, 2011). Cotton biotechnology has tremendous commercial implications. It can change the way cotton is cultivated. Cotton was one of the first genetically modified crops to be commercially released (Jones et al., 1996; Wilkins et al., 2005). To obtain high yields, several methods have been used by farmers to minimize the major threats of cotton. For example, weeds have been controlled traditionally by mechanically uprooting methods Weeds compete with crop plants and reduce the yield in both quantity and quality (ICAR, 2009). The implements used for mechanical weed control shear and tear the surface of the soil, resulting in the uprooting of plants. The introduction of herbicide-resistant crops has dramatically changed weed management in crop production systems (Owen, 2001). 5-enolpyruvylshikimic acid-3-phosphate synthase (EPSPS)-encoding bacterial genes transformed into crop plants by the use of stable genetic transformation can confer glyphosate resistance (Fitzgibbon and Braymer, 1990; Padgette et al., 1991).

Similarly, a gene of *Bacillus thuringiensis* (Bt), which is a Gram-positive bacterium, can be transformed into cotton for the production of crystalline protein (Schnepf et al., 1998), which is toxic to the larvae of different orders of insects e.g., Lepidoptera, Coleoptera, and Diptera. Genes of this sort are widely used to develop insect resistance in various crops (Gasser and Fraley, 1992; Ali et al., 2014). Among these genes, transgenic cotton that expresses insecticidal proteins from *B. thuringiensis* has been one of the most rapidly adopted GM crops in the world (Barwale et al., 2004; Dong et al., 2005; James, 2013; Qamar et al., 2015a,b). Transgenic cotton contains one or more Cry genes, such as Cry1Ac, Cry1Ac, and Cry2Ab, or Cry1Ac and Cry1F. These genes used in this transgenic cotton, also called Bt cotton, are considerably effective in controlling Lepidopteran pests, and the use of this cotton is highly beneficial to the grower and the environment by reducing the use of chemical insecticide sprays and preserving the population of beneficial arthropods (Carpenter and Gianessi, 1999; Tabashnik et al., 2009). In this study, the transformation of two Bt genes (Cry1Ac and Cry2A) and an herbicide resistant gene (cp4 EPSPS) into the local cotton variety VH 289 was attempted with the help of the *Agrobacterium* mediated transformation method. The constitutively expressed CaMV 35S promoter was attached to the Bt genes and the herbicide resistant gene during cloning, and this promoter was used for expression of these genes in cotton plants. These constructs were developed prior to the transformation at the Centre of Excellence in Molecular Biology (CEMB). This construct was created to develop the glyphosate tolerance gene (GTGene) for herbicide tolerance and an insecticidal gene (Cry1Ac and Cry2A) in the cotton variety VH 289. This promoter is an excellent candidate to drive strong and consistent expression of the transgene. This expression is restricted to the green tissues of plants. Thus, in the roots of the plant, there is no expression of these genes. Ultimately, based on the biosafety point of view, this transformation would not cause harm to soil microorganisms or the environment.

## Materials and methods

### Bacterial strains and plasmids

*E. coli* strains were grown at 37°C in LB medium using kanamycin selection. These bacterial cells were made competent using CaCl_2_. The plasmid harboring the Cry1Ac and Cry2A genes were transformed into the strains according to the heat shock method (Sambrook et al., 2006). Resistant colonies were selected on LB media with kanamycin selection. The plasmid was extracted from these cells following the standard protocol of The FavorPrep™ Plasmid Extraction Kit. We transformed the plasmid into *Agrobacterium tumefaciens* (strain LB4404) competent cells using a Bio-Rad Gene Pulser Electroporator Model 1652078, and 2 μl of the plasmid DNA vector was added to 10 μl of the bacterial cells. Cells were allowed to grow in YEP media for 1–2 h, and 50 ml of culture was spread onto YEP plates with antibiotic resistance.

### Transformation of plasmid into *Escherichia coli* and plasmid isolation

The Cry1Ac and Cry2A (Rao et al., 2011) plasmid along with the GTGene (Bajwa et al., 2013; Qamar et al., 2015a,b) plasmid were transformed into competent DH5α cells using heat shock. After transformation, 1 ml of SOC solution or LB broth was added, and the culture was placed in a shaker for 1–2 h at 37°C. Resistant colonies were selected again on kanamycin selection LB medium. The plasmid was isolated from a single isolated colony and was performed by following a standard protocol of phenol-chloroform-isoamyl alcohol (PCI) (Zasloff et al., 1978) with several modifications

### Electroporation of the Cry1Ac+Cry2A and GTGene plasmid into *Agrobacterium tumefaciens*

The plasmid was transformed into *A. tumefaciens* competent cells by using a Bio-Rad Gene Pulser Electroporator Model 1652078. The standard protocol (Mersereau et al., 1990) was used with minor modifications, and 2 μl of plasmid DNA was used to transform 10 μl of *A. tumefaciens* strain LB4404 cells. The transformed *Agrobacterium* cells were spread on YEP medium plates with kanamycin selection using 50, 100, and 150 μl of the cells. The YEP medium plates were incubated at 30°C for overnight.

### Confirmation of electroporation through colony PCR

The next day, resistant colonies were picked using toothpicks. Five colonies were randomly selected and dissolved in 1.5 ml tubes containing 50 μl of lysis buffer. The solution was heated at 95°C for 10 min. After centrifugation at 13,000 rpm for 10 min, colony PCR was performed.

### Primer designing

Reverse and forward primers were designed for the Cry2A gene and the GTGene gene using Primer3, a freely available primer design program. The detailed primer information is in Table 1 (Supplementary Figure 1).

**Table 1:** **Germination, tissue culture, and plant formation response of local cotton varieties**.

**Sr. No**.	**Varieties**	**Germination (%)**	**Plant formation (%)**
1	CIM-446	86	88
2	CIM-473	90	96
3	CIM-482	86	87
4	CIM-497	78	80
5	CIM-499	80	80
6	NIAB-78	90	89
7	NIAB-846	80	79
8	VH-289	95	98
9	MNH-147	77	75
10	MNH-786	89	97
11	BH-79	70	78
12	BH-95	81	81
13	BH-75	77	80
14	BH-118	60	90
15	VH-290	71	89

### Transformation of Cry1ac, Cry2a and, *GTGene* in cotton *(Gossypium hirsutum) var. VH-289*

#### Screening of different varieties

On the basis of seed germination, twelve varieties of cotton (*Gossypium hirsutum*) were used for screening. These varieties were VH-281, VH-289, BH-79, BH-75, BH-118, BH-95, MNH-786, CIM-446, CIM- 482, CIM-473, CIM-497, and NIAB-846. The local cotton variety VH-289 was selected for transformation because of its higher percentage of germination and greater resistance against CLCuV than other cotton varieties.

#### Delinting of seeds

Concentrated sulphuric acid was used to delint VH-289 cottonseeds at the rate of 100 ml/kg of cottonseeds. Cottonseeds of VH-289 were added to a glass beaker, and concentrated sulphuric acid was mixed in with the seeds. After adding H_2_SO_4_, the seeds were continuously stirred using a spatula for 10–15 min until the surface of seeds became shiny. To remove the acid completely, the seeds were thoroughly washed 5–6 times with tap water. At that time, floater seeds were separated from sinker seeds.

#### Germination index of VH 289

To determine the germination index of the VH 289 local cotton variety seeds, sterilized petriplates, and autoclaves filter papers were used. Autoclave filter paper was spread on sterilized petriplates, and 1 ml of autoclaved water was spread on the filter paper to soak the cotton seeds. Thirty cotton seeds of the VH 289 variety were spread on each petriplate, and the petriplates were covered with lids. The petriplates were incubated in the dark at 30°C for 48 h. The germination index of the VH 289 local cotton variety was calculated by using the following formula:

$$\begin{array}{l} GerminationIndex=\frac{GerminatedSeed}{TotalSeeds}\times 100 \end{array}$$

#### Sterilization and soaking of seeds

The delinted seeds of the VH-289 variety were sterilized using autoclaved 1 L Erlenmeyer flasks and autoclaved sterilized water. The cottonseeds of the VH-289 variety were sterilized by adding 1 ml of 10% SDS and 2 ml of 5% HgCl_2_ to the seeds. The cottonseeds were soaked by putting 500 seeds in an autoclaved flask with 100 ml distilled autoclaved water. This flask was covered with black paper and incubated in the dark at 30°C for overnight germination.

#### Embryo isolation

The next day, the testa of the cottonseeds was removed with forceps, and a surgical blade was used for the excision of the cotyledonary leaves. The mature embryos of the germinating seeds of the cotton variety were isolated using forceps and surgical blades. The isolated embryos were kept on moist filter paper prior to the transformation experiment.

#### Medium preparation

MS (Murashige and Skoog, 1962) broth was used to culture the transformed embryos with 50 mg/mL of kanamycin and 250 μg/ml of cefotaxime for selection.

#### Bacterial inoculum preparation

The *Agrobacterium* strain LBA4404 containing the Cry2A and GTGene individual plasmids was streaked on solidified agar medium containing kanamycin at 50 μg/ml and incubated for 24–48 h at 28°C. Single colonies were picked and inoculated in 10 mL of YEP broth containing 50 mg/mL of kanamycin in a 50 ml culture tube. The samples were incubated on a rotary shaker at 28°C for 24 h while shaking at 200 rpm. After centrifugation, the supernatant was discarded, and the pellet was resuspended in 10 ml of MS broth.

#### Shoot apex method

The *Agrobacterium* mediated genetic transformation of the mentioned local cotton cultivars was achieved using the shoot apical meristems isolated from the seedlings as explants. *A. tumefaciens* has synthetic genes for encoding the GTGene, CrylAc, and Cry2A genes, which were transformed into the cotton cultivars. The procedure was followed as suggested by Gould and Magallanes-Cedeno (1998).

A total of 6205 embryos from the *Cry1Ac* and *Cry2A* group and 6205 *GTG* embryos were used in the transformation experiments. After bacterial inoculum treatment, the culture was removed, and the embryos were shifted to MS medium plates without a selection drug. They were cocultivated for 3 days. The MS medium plates were kept in a growth room at a temperature of 25 ± 2°C with a photoperiod of 16 h of light and 8 h dark.

#### Selection on antibiotic medium

After 3 days (72 h) of cocultivation, the plantlets from the petriplates were shifted to test tubes containing the selection drugs kanamycin (50 mg/ml) and ceftriaxone (250 μg/ml). Different growth hormones, such as indole acetic acid (1 mg/ml) and gibberellic acid (1 mg/ml), were also added to medium for better growth of the transformed plantlets. The control plants were maintained on simple MS media. Every 15 days for a total of 2 months, the plantlets were subcultured. The transformation efficiency was calculated after 8 weeks of transformant growth on the selection medium (50 mg/ml kanamycin).

#### Establishment of transformed plants in soil

After 8–10 weeks, the plantlets were transferred to sigma pots containing sterilized compost soil that consisted of a mixture of equal proportions of clay, sand and peat moss (1:1:1). The plants were covered with plastic bags to maintain proper humidity and were kept in a growth room at a temperature of 25 ± 2°C and a photoperiod at 16 h light and 8 h dark. After 4 weeks, these plants were acclimated under sunlight and then shifted to a green house or field conditions for better growth.

#### Molecular analysis of putative transgenic cotton plants at T_0_ generation

The putative transgenic cotton plants with two Bt genes (Cry1Ac and Cry2A) and one GTG gene were analyzed by using high throughput molecular techniques. Extraction of high-quality genomic DNA from *Gossypium* (cotton) species was extracted by following the method suggested by Dellaporta et al. (1983) with minor adjustments.

#### Statistical analysis of transgenic cotton plants

Analysis of variance (ANOVA), least significant difference test (LSD), and Dunnett's test were performed to calculate the difference in insect mortality between control and transgenic plants.

## Results

### Genomic DNA extraction of putative transgenic cotton plants at T_0_ generation

For the extraction of the genomic DNA from the transgenic cotton plants, newly germinated leaves of the putative transgenic cotton plants were placed in liquid nitrogen and grounded into a fine powder. The powder was transferred into Eppendorf tubes, and 700 μl extraction buffer was added. The Eppendorf tube was incubated in a water bath at 65°C for 1 h. After incubation, an equal volume of chloroform:isoamylalcohol (24:1) was added. The solution was mixed completely with continual vortexing. After mixing, the solution was centrifuged at 13,000 rpm for 10 min. The supernatant was transferred to a new Eppendorf tube, and the pellet was discarded. Ice cold 60% isopropanol (v/v) was added, and the Eppendorf tube was incubated at −20°C for overnight until the DNA aggregated. The next day, the DNA pellet was harvested using centrifugation. The supernatant was removed, and the DNA pellet was washed with 1 ml of washing solution (80% ethanol+15 mM ammonium acetate) and centrifuged again for 15 min at 13,000 rpm (Supplementary Figure 1). After centrifugation, the genomic DNA pellet was dried, and it was suspended in low EC or PCR water. Agarose gel electrophoresis was used to estimate the concentration of the genomic DNA of the putative transgenic cotton plants.

### Polymerase chain reaction (PCR) of putative transgenic cotton plants at T_0_ generation

Newly germinated leaves of the transgenic cotton plants were used for the isolation of their genomic DNA, according to the protocol described by Dellaporta et al. (1983) with some modification. PCR was performed to detect the Cry1Ac and Cry2A genes and the GTG gene in the transgenic cotton plants. The extracted DNA from untransformed plants was used as a negative control and that of plasmid pk2Ac as positive control. PCR of Bt genes was performed using the following condition: 94°C for 5 min, 94°C for 45 s, 51°C for 45 s for Cry1Ac+Cry2A or 60°C for 45 s for GTG and 72°C for 45 s repeated 35 times (Supplementary Figures 2, 3). The amplified PCR fragments were evaluated on a 1% agarose gel and observed under UV light for the amplification of the genes with the specific primers found in Table 2 (Supplementary Figure 1).

**Table 2:** **Germination and tissue culture of cotton embryos for transformation**.

**Sr. No**.	**No. of embryos isolated**	**Agro-bacterium treated**	**MS plates**		**Selection tubes**		**Pots**	**Died**	**Shifted to green house**
			**Survive**	**Dead**	**Survive**	**Died**			
1	679	679	198	481	5	193	5	4	1
2	591	591	133	458	9	124	9	8	1
3	588	588	171	417	12	159	12	10	2
4	650	650	151	499	8	143	8	7	1
5	589	589	77	512	6	71	6	5	1
6	675	675	132	543	10	122	10	8	2
7	590	590	79	511	4	75	4	4	0
8	669	669	190	479	11	179	11	8	3
9	596	596	61	535	3	58	3	3	0
10	578	578	32	546	6	26	6	4	2
Total	6205	6205	1224	4981	74	1150	74	61	13

The primer sequences used for the amplification of the glyphosate resistance gene can be found in Table 3 (Supplementary Figure 1).

**Table 3:** **Efficiency of transformation**.

**Sr. No**.	**Number of experiments**		**Plants obtained after 3–4 weeks (selection medium)**	**Transformation efficiency**
**1**	**No. of embryos isolated for transformation**	**Control**	**Putative transgenic cotton plants**	**Survival percentage of experiments**
Total	6205	**100**	74	1.19%

### Bradford assay of putative transgenic cotton plants at T_0_ generation

A Bradford assay was conducted to quantify the total crude protein of the plants. Fresh leaves of the transgenic cotton plants were ground in liquid nitrogen with a mortar and a pestle to make a fine powder. Then, 600 μl of protein extraction buffer was added to an Eppendorf tube containing the fine powder, and the tube was incubated at 4°C overnight. The next day, the crude protein was isolated using centrifugation. After centrifugation, the supernatant was removed, and 780 μl of 1X PBS solution was added to the Eppendorf tubes containing the supernatant. Then, the Eppendorf tubes were incubated at room temperature for 10 min. Using a spectrophotometer, the amount of crude protein was calculated in transgenic cotton plants.

### Quantification of *Cry1Ac* and *Cry2A* endotoxins and *GTG* proteins of putative transgenic cotton plants at T_0_ generation

Enzyme linked immunosorbent assay (ELISA) was used to quantify the expression of all Cry1Ac, Cry2A, and GTG genes, according to the protocol provided by the Envirologix Kit (Cat # 051) temporally as well as spatially. Leaf samples of the transgenic plants were ground into a fine powder in liquid nitrogen, and 600 μl of the protein extraction buffer was added to the powder. Then, the mixture was incubated in an Eppendorf tube on ice for 1 h and then centrifuged for 25 min at 13,000 rpm. For further analysis, the supernatant was used for the quantification of the endotoxins by plotting the absorbance values of Cry2A and GTG test samples on the standard curve. The values for the expression of the endotoxin proteins were measured in ng per gram. The procedure used for the ELISA is as follows: 100 μl of the negative control was added to the ELISA plate. 100 μl of the calibrating sample and 100 μl of each diluted sample of the transgenic cotton plants were added to their respective wells. The contents of all wells were thoroughly mixed using a strip holder in a rapid circular motion for 20–30 s. The wells of the ELISA plate were covered with parafilm, and the samples were incubated for 15 min at ambient temperature. After the incubation, 100 μl of DNA-enzyme conjugate solution was added to each well. With the help of the strip holder, the contents of all the wells were thoroughly mixed. The wells were covered with parafilm and incubated for 1 h at room temperature. The ELISA plate was washed thoroughly with washing buffer. After washing, the ELISA plate was dried under air. Then, 100 μl of the substrate solution was added to each well, and the contents of the wells were mixed thoroughly. After mixing, 100 μl of the stop solution was added to each well and mixed thoroughly. Again, the ELISA plate was subjected to incubation. Finally, the ELISA plate was read at 450 nm to calculate the protein expression.

### Biotoxicity assay of putative transgenic cotton plants at T_0_ generation

A biotoxicity assay was used to check the efficacy of the endotoxins against the targeted insect pests of cotton, such as *Heliothis* larvae (2nd instar). In the procedure of the biotoxicity assay, five leaves from the upper, middle, and lower portion of the transgenic and control cotton plants were taken after 30, 60, and 90 days of crop age. Moist filter paper was placed in petriplates, and all the leaves were kept on separate petriplates. These transgenic and control cotton leaves were used to feed 2nd instar larvae of *Heliothis*. After 2–3 days, the mortality rate was noted, which was variable. The mortality range was 60–100%, while it was 0% in the control VH-289. The mortality rates were calculated as follows:

$$\begin{array}{l} \text{\%Mortality}=\frac{\text{No}.\;\text{of}\;\text{dead}\;\text{larvae}}{\text{Total}\;\text{no}.\;\text{of}\;\text{larvae}}\times 100 \end{array}$$

### Spray assay of putative transgenic cotton plants at T_0_ generation

A glyphosate spray assay was used for the confirmation, integration, and expression of the GTG gene (glyphosate resistance) in transgenic cotton plants of the VH-289 variety. During field growth, a glyphosate herbicide was sprayed on the transgenic and control cotton plants at the concentration of 1600 ml/acre in order to check the resistance. The glyphosate herbicide spray was also sprayed on weeds that were present in the transgenic cotton field.

### Plasmid constructs of Cry1Ac+Cry2A and GTG

**Figure 1:**
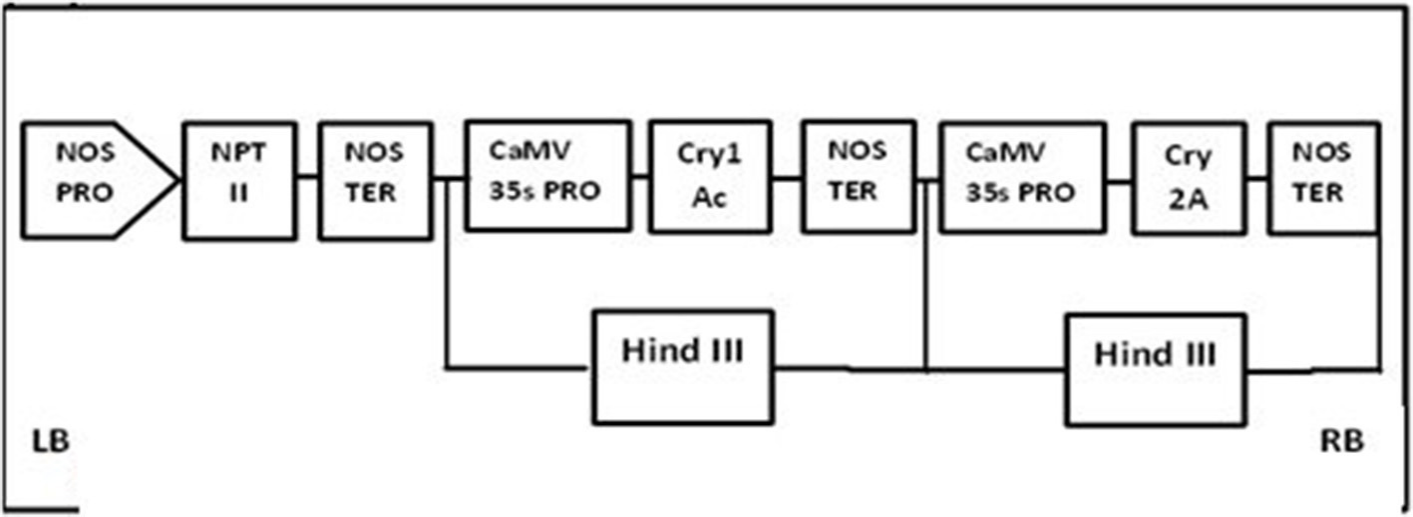
**Physical maps of CrylAc+Cry2A construct**.

**Figure 2:**
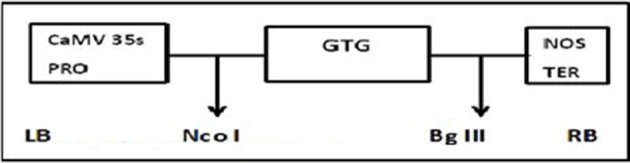
**Physical maps of GTG-gene construct**.

The plasmid construct for the Cry1Ac and Cry2A genes was cloned using HindIII into the pKHG4 plant expression vector with the 35S promoter, and the GTGene (codon optimized cp4EPSPS) was cloned using Nco1 and BglII into the pCAMBIA 1301 vector with the 35S promoter. These vectors were taken from the CEMB repository for transformation into cotton. The cassette maps of both constructs are shown in Figures 1, 2.

### Selection of cotton variety for transformation

Fifteen local varieties of cotton were screened for their ability to germinate and form mature embryos. Different regeneration responses were obtained from these varieties as shown in Table 1. The response was limited to only shoot formation in some varieties. Because of arrested growth, in these varieties, the plants starts dying at this stage. Thus, they could not produce roots on MS medium. Some shoot apices grew well with profound development on the MS medium and had root formation after 3–4 weeks. The plants regenerated from these varieties were shifted to the soil after 5 weeks, and their growth response in the soil was evaluated. The cotton varieties in which plants showed maximum shoot regeneration and the best adaptation to soil after their culturing were selected for the transformation experiments. Among these varieties, the response of VH 289 was found to be best. Thus, the VH 289 variety was selected for the transformation of Cry1Ac+Cry2A (pK2Ac) along with GTGene (Table 1).

### The transformation of pK2Ac and GTGene gene in cotton variety VH-289

**Figure 3:**
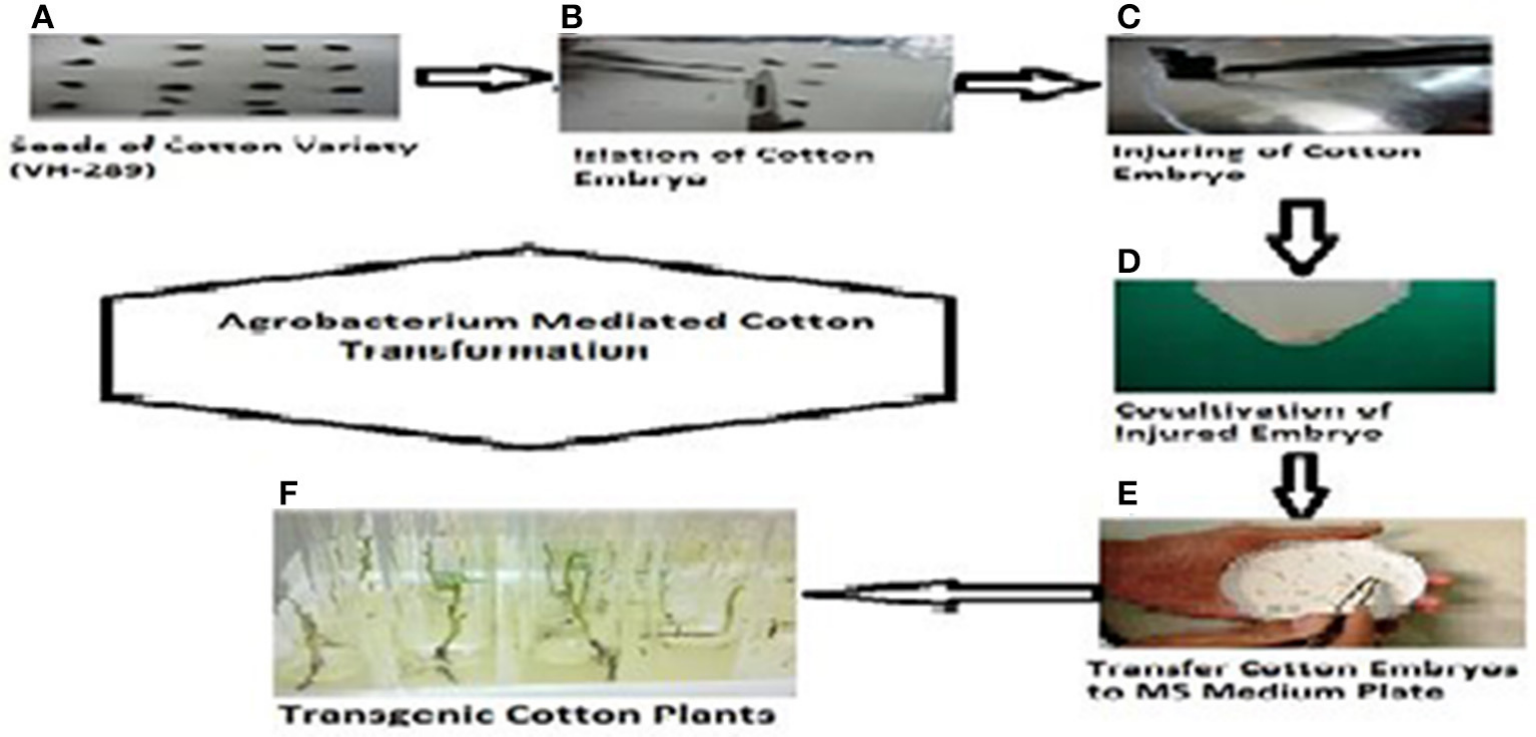
**Complete protocol of Agrobacterium mediated transformation in cotton**. **(A)** Germinated Seeds of VH-289, **(B)** embryo isolation, **(C)** injuring of cotton embryos with sharp blade, **(D)** co-culturing of injured embryos in MS broth with gene of interest (Cry2A and GTG), **(E)** cotton embryos are shifted to selection free MS medium plate, **(F)** cotton embryos shifted to test tubes with selection drug (kanamycin) for screening.

The *A. tumefaciens* strain LBA 4404 containing the pK2Ac (Cry1Ac+Cry2A) and GTG gene (cp4EPSPS) plasmids was used for the transformations. The vector construct containing the pK2Ac (Cry1Ac+Cry2A) genes driven by a CaMV35S promoter with the *nptII* gene as the selection marker, which confers resistance to kanamycin, was transformed into the cotton variety VH-289 by using the CEMB optimized embryo cut method (Rao et al., 2011). Similarly, the vector construct containing the Glyphosate Tolerant Gene (cp4EPSPS) driven by the CaMV35S promoter was also transformed into the above mentioned variety as a separate cassette. The harvested *Agrobacterium* culture containing the gene of interest was dissolved in MS broth. A total of 6205 embryos were used in the individual transformations of the pK2Ac and GTG genes. The putative transgenic cotton plants with both genes were screened using the selection medium containing 50 mg/mL of kanamycin (Figure 3, Supplementary Figure 1). Seventy-four plantlets were obtained after 8 weeks of selection (Table 2). The transformation efficiency was found to be 1.19% (Table 3). The plantlets were shifted to the selection free medium in test tubes for normal growth after one and half months. The putative transgenic cotton plants, which regained their roots and shoots, were shifted to loamy soil pots. The stable putative transgenic cotton plants that contained the pK2Ac and GTG genes were subjected to further molecular analysis. A schematic diagram of the transformation events are shown here in Figure 3, Supplementary Figure 1.

### Establishment of putative transgenic plants in the soil

The plants that survived the one and half month selection pressure of kanamycin and glyphosate were shifted to soil pots to acclimate. Thirteen putative transgenic cotton plants out of 74 shifted plants established growth in the soil pots (Table 2).

### Molecular analysis of putative transgenic cotton plants in T_0_ generation

#### Confirmation of CryIAc+Cry2A and GTGene with polymerase chain reaction

**Figure 4:**
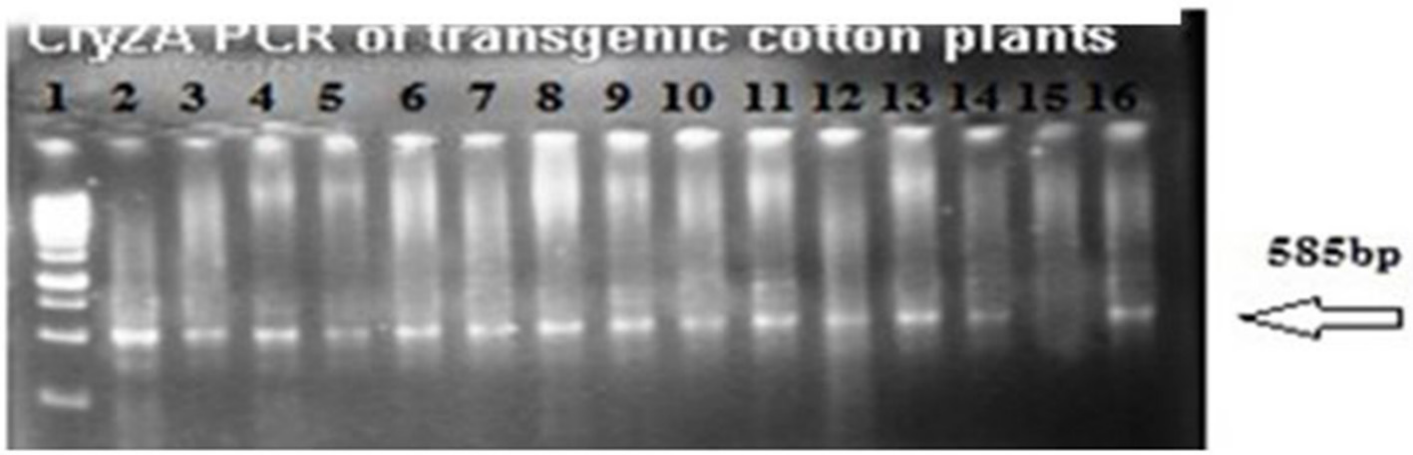
**PCR amplification of transgenic cotton plants of VH 289 for Cry2A**. Lane 1 represents 1 kb ladder; Lane 2 to 14 represent the transgenic cotton plants with Cry2Ac gene; Lane 15 shows negative control; Lane 16 is the positive control cotton plants.

**Figure 5:**
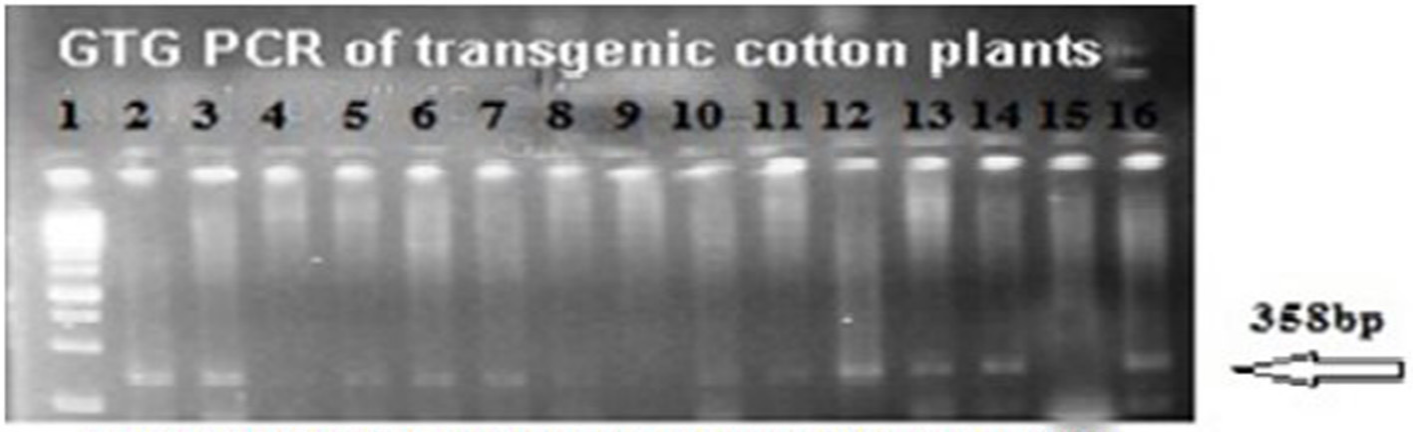
**PCR amplification of transgenic cotton plants of VH 289 for GTG-gene**. Lane 1 represents 1 kb ladder; Lane 2–14 represent transgenic cotton plants with GTG gene; Lane 15 represents negative control; Lane 16 represents positive control.

The putative transgenic cotton plants of the VH 289 cotton variety were confirmed to contain the transformed genes through PCR amplification using the gene specific primers for Cry2A and GTGene (Figures 4, 5). An amplification product of 585 bp was found for the Cry2A gene, and a product of 358 bp was found for the GTGene gene. It is clear from Figure 4 that the Cry2A gene was integrated into the genome of all thirteen (13) putative transgenic cotton plants. Figure 5 depicts the amplification of the 358 bp product for the GTG gene in cotton plants #1–13. No amplification was seen for plants #4, #8, and #9, as shown in Figure 5 and Table 4, which shows a summary of the confirmation of the transgenes in cotton plants.

**Table 4:** **Summary of PCR amplification of putative transgenic cotton plants**.

**Sr. No**	**Plants name**	**GTG**	**Cry2A**	**Description results of PCR**
1	VH 289 (2)	+ve	+ve	Positive for Bt and herbicide resistance gene
2	VH 289 (18)	+ve	+ve	Positive for Bt and herbicide resistance gene
3	VH 289 (25)	+ve	+ve	Positive for Bt and herbicide resistance gene
4	VH 289 (34)	−ve	+ve	Positive for Bt, negative herbicide resistance gene
5	VH 289 (52)	+ve	+ve	Positive for Bt and herbicide resistance gene
6	VH 289 (53)	+ve	+ve	Positive for Bt and herbicide resistance gene
7	VH 289 (55)	+ve	+ve	Positive for Bt and herbicide resistance gene
8	VH 289 (57)	−ve	+ve	Positive for Bt, negative herbicide resistance gene
9	VH 289 (64)	−ve	+ve	Positive for Bt, negative herbicide resistance gene
10	VH 289 (66)	+ve	+ve	Positive for Bt and herbicide resistance gene
11	VH 289 (69)	+ve	+ve	Positive for Bt and herbicide resistance gene
12	VH 289 (72)	+ve	+ve	Positive for Bt and herbicide resistance gene
13	VH 289 (73)	+ve	+ve	Positive for Bt and herbicide resistance gene
15	Negative control	*−ve*	*−ve*	Non transgenic control
16	Positive control	+ve	+ve	Positive transgenic control

#### Enzyme linked immuno-sorbent assay (ELISA)

**Figure 6:**
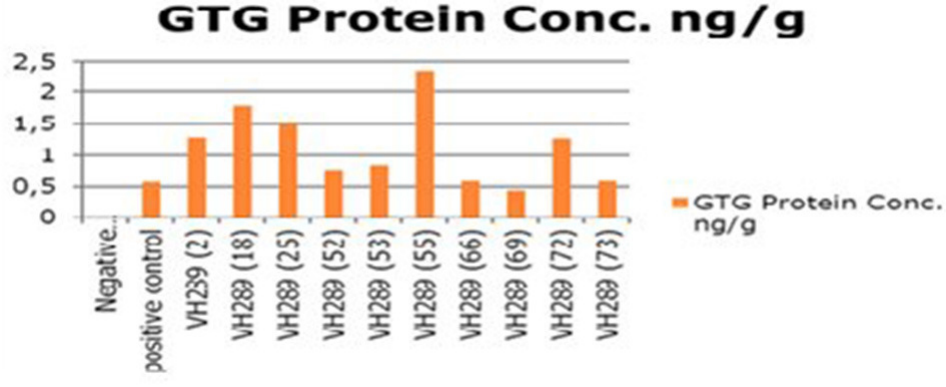
**Concentration of GTG protein of transgenic cotton plants at T_0_ generation**. Lane 1 represents control cotton plant; Lane 2 shows positive control; Lane 3–12 represent cotton plants.

**Figure 7:**
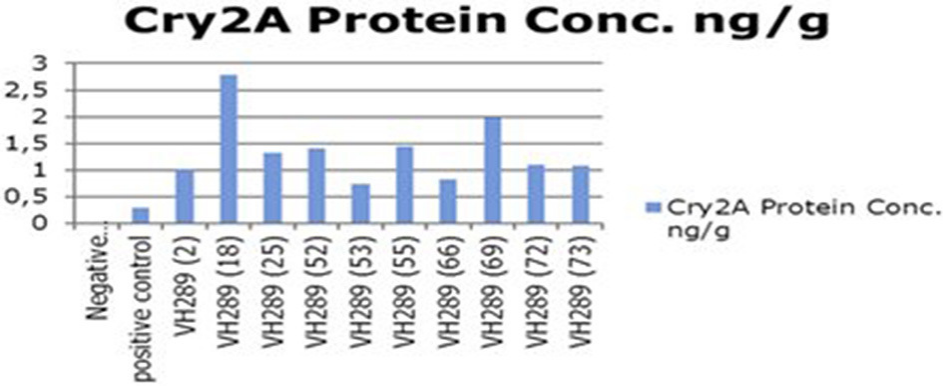
**Concentration of Cry2A protein of transgenic cotton plants at T_0_ generation**. Lane 1 represents control cotton plant; Lane 2 shows positive control; Lane 3–12 represent cotton plants.

The ultimate objective of the current transformation experiments was to express the Cry2A and GTGene genes in the form of the Cry proteins and the GTG protein. An enzyme linked immunosorbant assay was used to screen the plants for the Cry2A protein (**Figure 7**) and the GTG protein (Figure 6). The total protein was isolated from 10 plants, and the amounts are as follows (mg/ml): VH 289 (2), VH 289 (18), VH 289 (25), VH 289 (52), VH 289 (53), VH 289 (55), VH 289 (66), VH 289 (69), VH 289 (72), and VH 289 (73). The protein samples of the positively amplified putative transgenic cotton plants were bound to the microtiter plate wells with specific antibodies for the Bt protein and the GTG protein, and the presence of the proteins was detected using a color reaction. The quantification of the BT and GTG proteins of 10 putative transgenic plants was performed using the Micro Plate ELISA Reader Model ELx800/. A total of 10 mg of the Cry2A protein per g of fresh leaves was obtained (Figure 7), while in the case of GTGene (Figure 6), 8 mg of the GTGene per g of fresh leaves was obtained as shown in Table 5.

**Table 5:** **Concentration Cry2A and GTG protein of putative transgenic cotton plants (T_0_ Generation)**.

**Sr No**	**Plants**	**GTG Protein Conc. ng/g**	**Cry2A Protein Conc. ng/g**	**Result of expression protein**
1	Negative control	0	0	Negative control
2	Positive control	0.58	0.302	Positive control
3	VH289 (2)	1.29	1.002	Plant have both GTG and Bt protein
4	VH289 (18)	1.79	2.79	Plant have both GTG and Bt protein
5	VH289 (25)	1.52	1.33	Plant have both GTG and Bt protein
6	VH289 (52)	0.75	1.41	Plant have both GTG and Bt protein
7	VH289 (53)	0.82	0.74	Plant have both GTG and Bt protein
8	VH289 (55)	2.36	1.45	Plant have both GTG and Bt protein
9	VH289 (66)	0.59	0.82	Plant have both GTG and Bt protein
10	VH289 (69)	0.44	1.98	Plant have both GTG and Bt protein
11	VH289 (72)	1.27	1.10	Plant have both GTG and Bt protein
12	VH289 (73)	0.59	1.09	Plant have both GTG and Bt protein

#### Biotoxicity leaf assay

To determine the efficacy of the Cry1Ac and Cry2A proteins in the transgenic plant leaves, the leaves were subjected to the insect larvae of *Heliothis* at different time intervals, e.g., 20, 40, 60, and 80 days. The results showed a mortality rate ranging from 60 to 100% for the transgenic plants, while in control plants, all larvae remained alive and showed progress in their weight. From these results, it is clear that the transgenic plants have the capability to kill the larvae of *Heliothis*. Thus, the toxin can kill the insect at an initial level (Table 6, Figures 9, 10).

**Table 6:** **Tabulation of bio toxic leaf assay of transgenic cotton plant (T_0_ Generation)**.

**Plants**	**Mortality % age of ***Heliothis*** larvae**			
	**20 days**	**40 days**	**60 days**	**80 days**
Control	0	0	0	0
VH 289 (2)	90	85	85	76
VH 289 (18)	100	90	85	80
VH 289 (25)	95	90	87	85
VH 289 (52)	80	80	77	75
VH 289 (53)	80	80	78	75
VH 289 (55)	90	85	85	70
VH 289 (66)	75	70	65	60
VH 289 (69)	100	90	67	80
VH 289 (72)	85	80	75	75
VH 289 (73)	85	80	75	70

**Figure 8:**
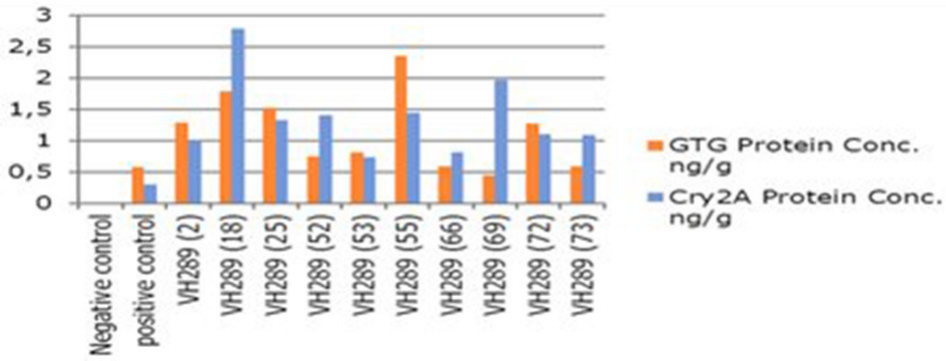
**Comparison concentration of GTG gene Cry2A gene protein of transgenic cotton plants at T0 generation**. Lane 1 represents control cotton plant, Lane 2 shows positive control, Lane 3–12 represent cotton plants.

**Figure 9:**
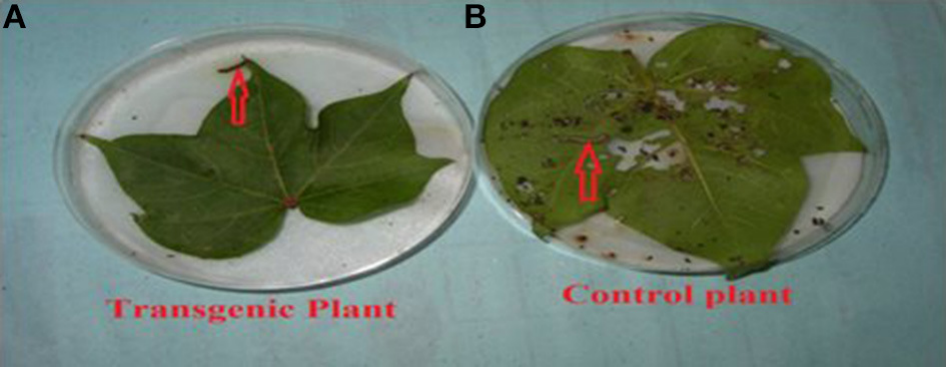
**Insect bioassay of leaves of transgenic and control cotton plant**. **(A)** Transgenic plant leaf showing killed larvae after eating a small portion of leaf and plate. **(B)** Control non-transgenic plant. Larva remained active and alive after damage.

**Figure 10:**
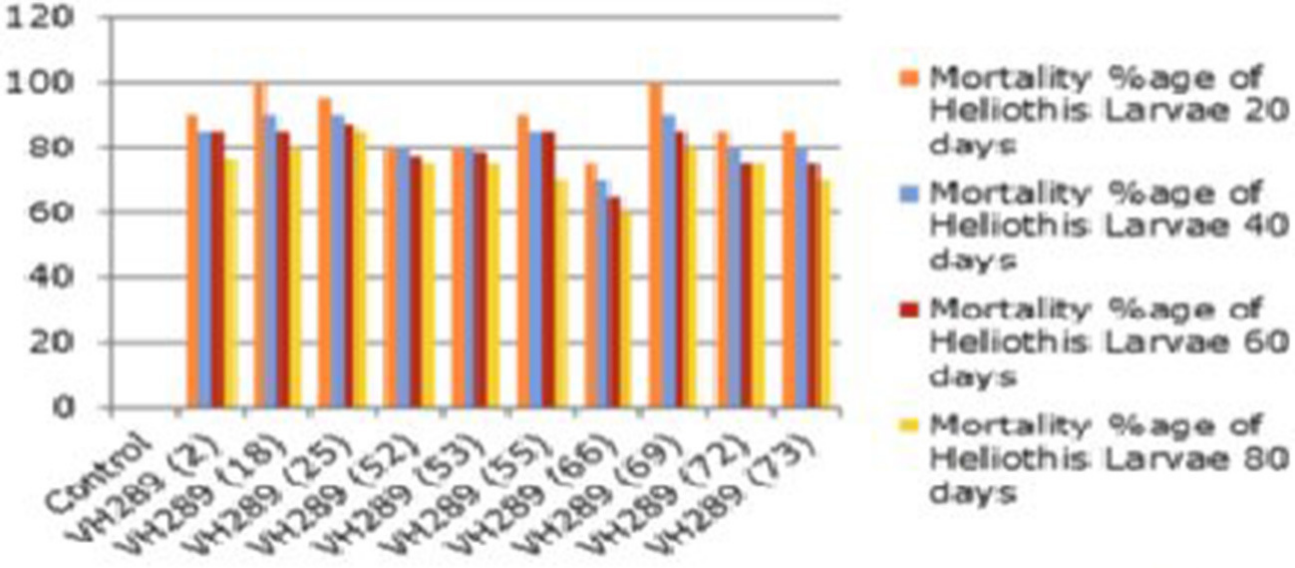
**Mortality percentage of american bollworm of putatire transgenic cotton plant at the time interval of 20, 40, 60, and 80 days at T_0_ generation**.

#### Herbicides spray assay (T_0_ generation)

**Figure 11:**
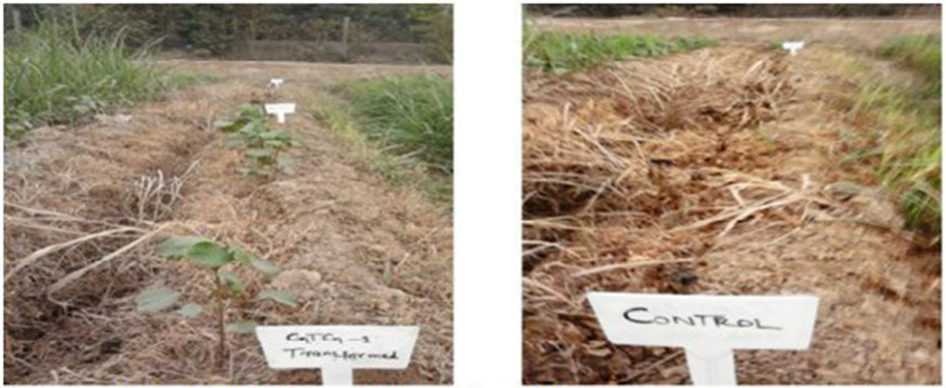
**Glyphosate spray assay of transgenic and control cotton plants**.

A glyphosate herbicide spray assay was used to determine the resistance of the transgenic cotton plants against glyphosate in field conditions at T_0_ generation. Plants were sprayed with Roundup Ready weedicide at a ratio of 1600 ml/acre, and after 5 days, 10 out of 13 plants remained alive and resisted the herbicide stress. The remaining three plants showed necrotic legions and dead tissues comparable to the dead weed plants (Figure 11). Figure 5 represents the glyphosate positive and control plant spray assay. The results of spray assay are tabulated in Table 7.

**Table 7:** **Glyphosate spray assay of transformed plants**.

**Serial No**.	**Plant name**	**Glyphosate spray concentration (ml/acre)**	**Results**
1	VH 289 (2)	1600	Plant survived and showed resistance
2	VH 289 (18)	1600	Plant survived and showed resistance
3	VH 289 (25)	1600	Plant survived and showed resistance
4	VH 289 (34)	1600	Plant died
5	VH 289 (52)	1600	Plant survived and showed resistance
6	VH 289 (53)	1600	Plant survived and showed resistance
7	VH 289 (55)	1600	Plant survived and showed resistance
8	VH 289 (66)	1600	Plant Resistant
9	VH 289 (57)	1600	Plant died
10	VH 289 (64)	1600	Plant died
11	VH 289 (69)	1600	Plant survived and showed resistance
12	VH 289 (72)	1600	Plant survived and showed resistance
13	VH 289 (73)	1600	Plant survived and showed resistance
14	Control	1600	Plant died

#### Determination of the copy no. and location of Cry2A and GTGene in cotton

**Figure 12:**
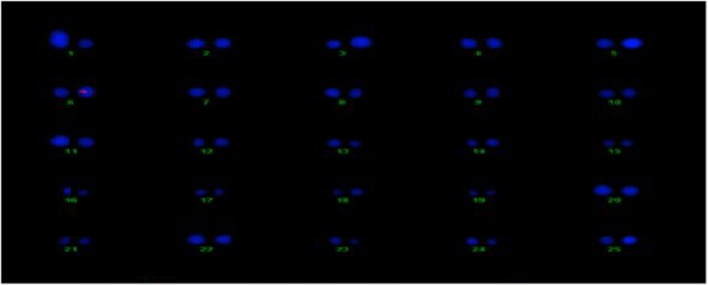
**Determination of Cry2A transgenic location on cotton chromosomes**.

**Figure 13:**
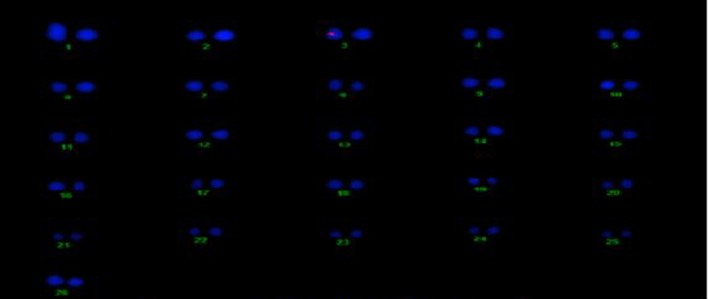
**Determination of GT-Gene transgene location on cotton chromosomes**.

The transgene copy no. and location of the Cry2A and GTGene genes was determined for the transgenic plant line VH 289 (55-4), which showed good expression of the Cry2A and GTGene proteins and resulted in a higher yield, by using gene specific probes. All transgenic plants for the VH 289 (55-4) line have shown signal in the nucleus at chromosome no. 6 for Cry2A and chromosome no. 3 for GTGene, but no signal was observed in the control plant (Figures 12, 13).

## Discussion

Cotton is a crop plant, and a high yield is the ultimate goal of crop plants. The major constraints for production yields of cotton are insect pests, weeds, and viruses. Our recent study proposed to control insect pests and weeds through the genetic modification of cotton by using the codon optimized Cry1Ac+Cry2A genes along with the cp4EPSPS gene. Bakhsh et al. carried out a similar study in 2014, in which the Cry1Ac gene was used through genetic transformation in cotton to control insects (Bakhsh et al., 2014). Deng et al. (2014) also carried out another similar study for weed control. It has been reported by Keller et al. (1997) that genetic modifications through the introduction of *Bt* and GTG genes are more beneficial for the improvement of crop plants against insects and weeds than conventional breeding methods, which are carried out by using successfully tissue culturing the anthers. Similarly, in 2009, Chawla produced this in parallel using protoplast culture (Chawla, 2009). Hence, the idea that producing genetically modified cotton plants with resistance to insects and weeds by simultaneously transforming the Cry1Ac+Cry2A gene along with the GTG-gene into Pakistan's local variety of cotton has been cemented.

In our current study, we used VH 289, a local variety, for genetic transformation. Bakhsh et al. (2012) used this local variety of cotton in his study in which the *Bt* gene was transformed for the infusion of insect resistance. We used the gene pyramiding approach to produce multiple traits in a single variety by introducing the two *Bt* genes, namely cry1Ac+cry2A (Rashid et al., 2008), and resistance to glyphosate (N-phosphonomethylglycine), commercially known as “Roundup.” This is the most widely used herbicide today. Glyphosate is a nonselective herbicide, capable of inhibiting the growth of crops and weeds with broad leaves (Schmid and Amrhein, 1995). We obtained 74 plants while processing 6205 embryos for the genetic transformation. Thus, 74 plants were considered putative transgenic plants, resulting in a transformation efficiency of 1.19%. A similar transformation efficiency (1.1%) has been reported for cotton plants by Lei et al. (2012) by using the shoot apex method for transformation. Rao et al. (2011) and Bajwa et al. (2013) also obtained parallel transformation efficiencies.

### Molecular analysis

After obtaining the putative transgenic plants, we subjected these 74 putative plants for molecular analysis. The molecular analysis data indicated that 10 out of the 74 putative transgenic cotton plants, VH 289 (2), VH 289 (18), VH 289 (25), VH 289 (52), VH 289 (53), VH 289 (55), VH 289 (66), VH 289 (69), VH 289 (72), and VH 289 (73), had positive amplification at T_0_ generation, for both the *Bt* genes (Cry1Ac and Cry2A) and the glyphosate resistant gene (GTG). Another molecular technique, ELISA, was used to quantify the transgenic proteins Cry1Ac+Cry2A and glyphosate (GTG) in plants. Total protein from all selected 10 transgenic cotton lines, namely VH 289 (2), VH 289 (18), VH 289 (25), VH 289 (52), VH 289 (53), VH 289 (55), VH 289 (66), VH 289 (69), VH 289 (72), and VH 289 (73), was isolated by following the protocol of Boopal et al. (2014) at the T_0_ generation. The ELISA for the quantification of the Cry2A and GTG proteins was done by using the AP 005 and AP 051. The Envirologix kits showed variation in the expression of the transgenic proteins. Cry2A and GTG expression was highest in the VH 289 line (18) at 2.79 ng/g and in the VH 289 line (55) at 2.36 ng/. The lowest expression was in the VH 289 line (53) at 0.74 ng/g and the VH 289 line (69) at 0.44 ng/g (Figure 8). The results of our study were in agreement with a study by Bakhsh et al. (2012). In total, 10 transgenic cotton plant lines at the T_0_ generation were found to be positive using PCR amplification, and these plants were also shown to produce significant quantity of transgenic proteins. It was also observed these transgenic plants were 10 times more resistance to insects and herbicide spray than control plants. Hence, it can be concluded, in view of our current results, transgenic cotton plants with both of the two *Bt* genes and one GTG gene can successfully be produced in the shortest possible time other than conventional breeding methods. These putative transgenic plants have a significantly desired resistance and are also environmentally friendly. Therefore, it can be said that the gene transformation method will be the main method used in the future for the production of genetically modified cotton plants.

### Biotoxicity leaf assay

The biotoxicity leaf assay was used to compare the efficiency of the transgenic proteins Cry1Ac and Cry2A in plants at different time interval with *Heliothis* (2nd instar larvae). For the biotoxicity assay, young leaves of the transgenic cotton plants were collected at the intervals 20, 40, 60, and 80 days of crop age. Then, they were subjected to a leaf bioassay. Three larvae were placed in each petriplate with a transgenic plant leaf inside in triplicate, along with a control, non-transgenic plant in a separate plate, as done by Kranthi et al. (2005). A study of transgenic protein toxin level titer is very crucial, as it must be in a sufficient quantity at the time of insect infestation to protect the crop against Lepidopterons, especially boll worms (Bakhsh et al., 2009). In the present study, the insect assay was performed to check the toxin level in the transgenic cotton plants at the T_0_ generation. The results clearly demonstrated that the level of Cry1Ac and Cry2A protein goes down, and the percentage of insect damage decreases with the passage of time, as the toxin level fall to the lowest levels, i.e., 0.5–3.0 ng/g in the T_0_ putative transgenic cotton plants. Comparable results were also reported by Ferré and Van Rie (2002), James (2004), Bakhsh et al. (2009), Manjunatha et al. (2009) and Adamczyk et al. (2009). ANOVA, LSD, and Dunnett's test also demonstrated that the difference of insect mortality between the control and transgenic plants was significant. In the whole plant insect bioassay, our studies showed the leaf damage differences in transgenic and control plant, and these results supported the point raised by Kranthi et al. (2005) and Gould and Magallanes-Cedeno (1998). In short, it can be concluded that the insect resistant cotton plants can be produced by transferring the two *Bt* genes (Cry1Ac and Cry2A). However, the level of the *Bt* transgenic proteins observed decreased with the passage of time. The biotoxicity assay showed that 74 putative transgenic plants have a considerable level of transgenic *Bt* proteins to protect them against insect attack. However, this decrease in the level of transgenic proteins was also noted in all 74 confirmed transgenic plants. This decrease might be because of either a weak promoter region or because of the aging process. It would be of interest in future research to determine how to maintain the level of transgenic *Bt* protein in the cells of transgenic cotton plant.

### Herbicides spray assay

Weeds are major constraints that cause damage to crop plants by competing for nutrients, space, and water. Conventional methods to control weeds, such as manual hoeing and the uprooting of weed plants, result in the loss of crop plant by unavoidable injuries. It is also a tedious process because it demands extra labor and consumes large amounts of time. Herbicide tolerance has consistently been considered a dominant trait of investigation since the beginning of transgenic plant commercialization (1996–2007). In 2007, 114.3 million hectares were used for biotechnological products cultivation. Sixty-three percent of which (72 million hectares) was dedicated to herbicide tolerant plants (James, 2007). It has also been noted that herbicides not only cause injury to weeds but are also damaging to crop plants. Commercially available herbicides are usually blind and unable to differentiate between weeds and crop plants. Thus, sometimes, they are equally lethal for both. Glyphosate kills plants by inhibiting the enzyme 5-enolpyruvylshikimic acid-3-phosphate synthase (EPSPS), which is necessary for the formation of the aromatic amino acids tyrosine, tryptophan, and phenylalanine. These amino acids are important for protein synthesis, thus linking the primary and secondary metabolism (Carlisle and Trevors, 1988). In view of this problem, it is important to produce herbicide resistant crop plants. In the present study, we transformed the GTG-gene, which is resistant to the herbicide glyphosate, along with the two other *Bt* genes mentioned. A glyphosate herbicide spray assay was used to determine the resistance of the transgenic cotton plants against glyphosate under field conditions at the T_0_ generation. Plants were sprayed with Roundup Ready weedicide at the concentration of 1600 ml/acre, and after 45 days, it was observed that 10 out of 13 plants remained alive and resisted the herbicide stress. The remaining three plants showed necrotic spot and dead tissues comparable to dead weed plants. Similar results were also presented by different researchers such as Carpenter and Gianessi (1999), Debora (1994), Gianessi et al. (2002), Mark (2013) and Ali et al. (2010). In view of the above results, it can be concluded that transgenic cotton plants that contain the GTG-gene have considerable resistance against glyphosate. This finding may be useful for the future engineering of cotton crops.

### Location of Cry2A and GTGene in cotton

FISH was preferred to Southern hybridization analysis in determining the copy number of the transgenic plants (Tsuchiya and Taga, 2001). The transgenic plants that showed good expression of the transgenic Cry2A and GTG-gene proteins were analyzed to determine their copy number of the genes as well as the chromosome location of their genes via FISH. This analysis is important because the transgene copy number and the location of transgene does matter for transgenic expression as determined by Rao et al. (2013), while determining the expression of the PhyB gene in transgenic cotton plants that had different copy numbers and locations. The results of this study coincide with the results of Rao et al. (2013). Hence, we conclude that the number of transgenes and their location does not affect the expression level of the transgene.

## Conclusion

The CEMB transgenic cotton lines harboring Cry1Ac+Cry2A along with the codon optimized cp4EPSPS gene (GTGene) showed significant resistance to Lepidopteran insects with 100% mortality of the insects. The lines also showed a considerable resistance against a broad spectrum of herbicides, with a tolerance limit of 1600 ml/acre. This ultimately leads to an increase in cotton yields. After advanced generation trials, the poor farmers of Pakistan can raise these selected transgenic cotton lines because they hold great potential. This study will help scientists with ideas for the future engineering of cotton crops.

### Conflict of interest statement

The authors declare that the research was conducted in the absence of any commercial or financial relationships that could be construed as a potential conflict of interest.
