# Supplementary figures and images for: Transformation and Evaluation of Cry1Ac+Cry2A and GTGene in Gossypium hirsutum L
Source: Front Plant Sci. 2015 Nov 12;6:943. doi: 10.3389/fpls.2015.00943 (PMC4641916; doi:10.3389/fpls.2015.00943)

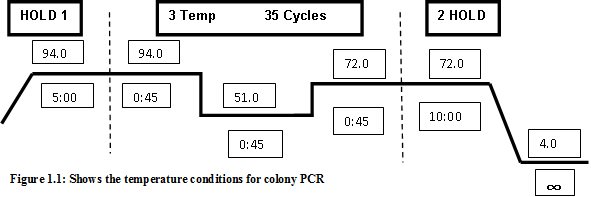

Supplement: Supplementary file 1 [file Image1.JPEG]

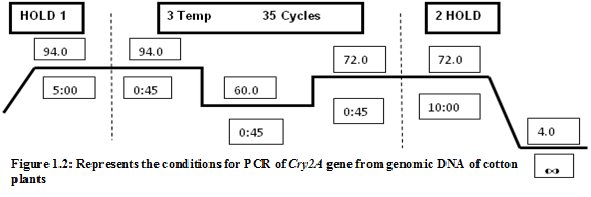

Supplement: Supplementary file 2 [file Image2.JPEG]

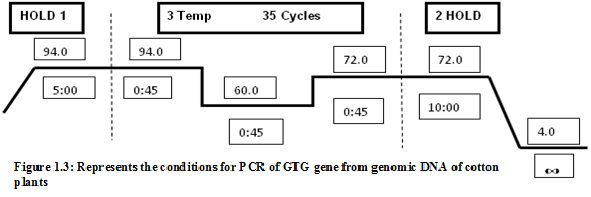

Supplement: Supplementary file 3 [file Image3.JPEG]
